# Treading & threading memories: a personal encounter with forest and people in Southern Venezuela

**DOI:** 10.1186/1746-4269-9-55

**Published:** 2013-08-06

**Authors:** Egleé L Zent

**Affiliations:** 1 Laboratory Human Ecology, Instituto Venezolano de Investigaciones Científicas, Panamericana km 11 Ado 20632, Altos de Pipe, edo. Miranda, Venezuela

**Keywords:** Ethnoecology, Ethnobotany, Fieldwork, Amazon, Jotï, Amerindian, Evocations

## Abstract

This brief essay, which represents the third editorial of the series "Recollections, Reflections, and Revelations: Ethnobiologists and their First Time in the Field", captures a few memories of the author's first fieldwork in the Venezuelan rainforest. It is a collage of objects, subjects, feelings, spaces, and events that pendulate in spheres of meaning straddling between the author's identities as both a student and a woman. The author's evocations of fifteen years ago are diluted in lasting reflections about what could be ethnoecology embraced by spaces of interactions and associations between organisms and their surroundings.

## Abstract (de)

**Resumen:**

Este breve texto, que representa el tercer editorial de la serie “Recuerdos, Reflexiones y Revelaciones: Etobiólogos y su Primera Salida de campo”, atrapa algunos recuerdos del primer trabajo de campo del autor en la selva amazónica venezolana. Constituye un entramado de objetos, sujetos, sentimientos, espacios y sucesos que pendulan en esferas de significados cabalgando entre las identidades del autor como estudiante y como mujer. Las evocaciones del autor de hace 15 años se diluyen en reflexiones que perduran acerca de lo que podría ser etnoecología abrazada por espacios de interacciones y asociaciones entre los organismos y sus entornos.

## 

Ethnoecology entrapped me by its fascinating eclectic and transdisciplinary qualities. Not just theoretical and applied initiatives are inherently articulated in ethnoecology, but also different epistemologies, events and life encounters, such as evocations [1]. These traits manifest themselves in the dynamics of selecting interesting problems, hypotheses and methods. Even the utterance of what ethnoecology means takes more than a few words when someone raises the simple question: what do you do? In a nutshell, ethnoecology was for me a good pretext to live a text and appreciate the contexts of how people and their environments interrelate. This paper hopes to provide a text about the context that gave meanings to my pretext to live among the Jotï, an Amerindian group of about 1000 persons scattered in Amazon and Bolívar States of the Venezuelan rainforest ([2,3] see http://ivic.academia.edu/Zent). Due to space limitations, here I explore just three key issues: (1) *articulation* of personal and academic life; *(2) alterity,* in a biocultural setting very different from where I was raised; and (3) *relativity* of notions, values and principles.

## Context

**Figure 1:**
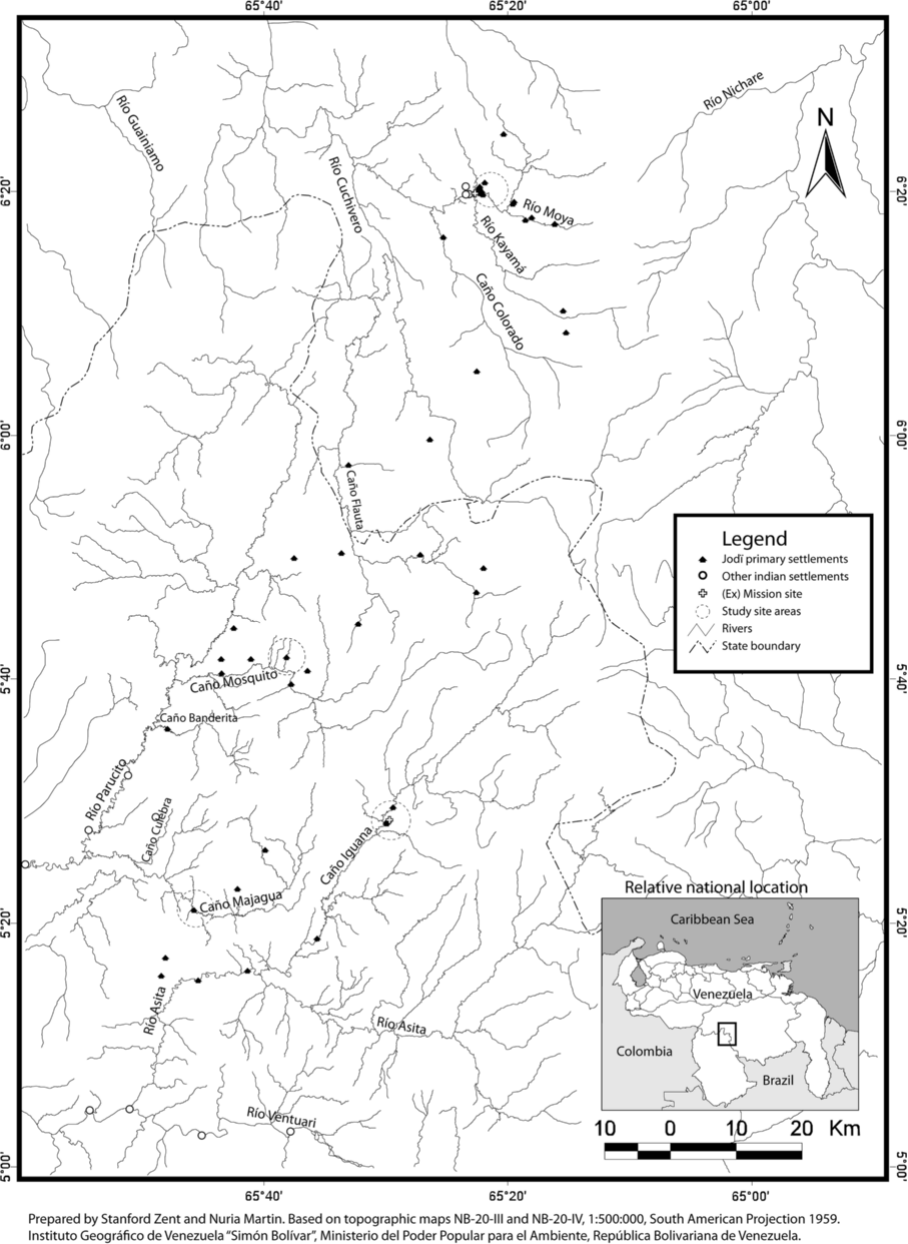
Jotï homeland and forested landscape, April 2012.

In April 1996, Stanford, my life and academic partner, and I, initiated a three-year long ethno-ecological fieldwork among the Jotï in their stretch of high forest territory located in the Sierra Maigualida (Figure 1). The Jotï, as well as their homeland, were at the time little known in the scientific community. Published reports of the Jotï appeared for the first time in 1969 [4-6], and some ethnological studies were conducted during the 70′s and 80′s [7-15]. By the mid 90′s however, just 11 references mentioned the Jotï. Likewise, the Sierra Maigualida had been explored mostly in the high tepuy areas whereas below 1500 m asl there was very little information available [16-19]. The virtual absence of previous ethnoecological and botanical literature provided a double stimulus for our study. From the outset, a main goal was to propose that a protected area be created for both the people and the mountain range. Thus, we expected to generate high quality data-bases to certify the Jotï’s property of their land through their fine traditional ecological knowledge. Our theoretical and methodological background was quite eclectic, ranging from Anthropology, Art History, Botany, Ecology, Linguistics, to Poetry. While both of us had previous field research experiences: I in the páramos, a unique beautiful ecosystem but a setting that was almost my homeland in the high tropical Andes above 3000 m asl; and Stanford among the Piaroa, in the Amazonian upland forests. I think his blood was already impregnated with the jungle. He loved the idea of going back to live among the trees. Different from him, I had to explore my own capability to live in the forest and with forest people. In this context, during the summer of 1994, Stanford and I set off for the Jotï territory to explore the (logistic, material, legal, spiritual) viability of conducting long-term field research there. We did not know where their homes were located. We knew that no highways or roads existed to go there by way of motor vehicles. We were not sure whether there was fluvial access to some communities but we were sure we could reach them on foot. This was the first time that I was looking for a site to live and carry out my own project in a totally new social and ecological setting. The memories below illustrate how we initiated our articulation with the Jotï and the Maigualida, a few of the many relative aspects of life, and my alterity space among my discrete *self* and the extreme *other* of those early days.

## Articulation

### June 1994

**Figure 2:**
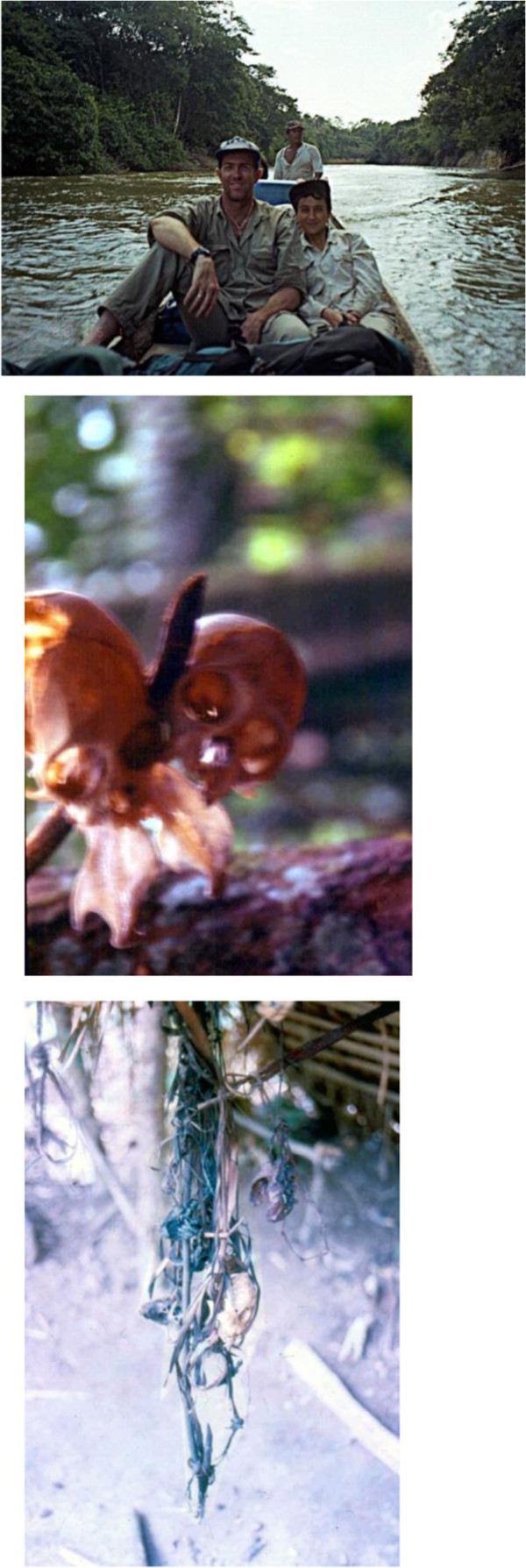
Jkalo Ijkuala: Jotï Children playing and facing the River and young Mother, May 2012b.

*The morning when we left San Juan de Manapiare in the dugout canoe in a blind search of Jotï communities has a soothing taste in my mind. We departed with Basilio and Joel, Piaroa and Yabarana friends of Stanford, who were experienced woodsmen and had just a global sense of where Jotï settlements could be found. The river seemed malleable and we advanced slowly. Humidity was everywhere. Rain is almost non-stop in this area from June to August. Thirsty plants welcomed the water without satiating the craving for more. The Manapiare gave way to the Ventuari River the first evening and we hung our hammocks for the night in a pleasant Yekwana settlement. However, the people there had very little to say about the Jotï. The next morning we reached the mouth of the Asita, a narrow tributary of the Ventuari. After a short ride, we unexpectedly came to the first Jotï community we had ever seen right on the riverbank. At home were three shy but friendly people, a young man in his 30′s, and two girls, a teenager probably 12 and a smiling one of around 9 years old. There was a single shelter and a little garden recently planted. They did not look suspicious but curious about us climbing their river* (Figure 2)*. They laughed at our many tries to communicate with them in Spanish, Piaroa, Yabarana or English. Still years after this first meeting we wondered why the Jotï did not have any recollection of contacts with other ethnic groups except the Eñepa even though their land is bordered by that of several ethnic groups. The Jotï seemed to have chosen to be un-contacted or to have few associations with these “other people”. We walked around the tiny village for about 40 minutes while the curious residents followed us, and then we were off again. Although no interchange of foods, goods or intelligible spoken words were possible, I spent the next hour thinking of many nonverbal ways to communicate and grasp life and their many meanings. We did not see these people again but met many of their relatives upriver. Several hours later, we left the main course of the Asita and entered the Jkalo Ijkuala (Caño Iguana). The waterway narrowed even more. Along the banks, I could see tangled plants of multiple sizes and colors lingering here and there, competing for light in a fearless survival quest. Above, the sky was cut by giant canopy trees unaware of time and silent. A soft chant was heard faraway. Then it happened, one of the most intense aesthetic experiences I ever had: flanked by deep forest through dark gentle waters, being followed by myriads of eyes and so many tones and shapes of green, unable to think… It was a non-rational touch with my archaic self and a non-canonic prettiness that somehow my blood, my cells, recognized as alike. It called me without words. It was difficult to focus my perception. The naked beauty and my own primitiveness scattered in the rain forest overwhelmed me.*

*Less than an hour later on the opposite bank we saw a couple of lean-to shelters. Our guides laughed, regarding them as feeble and grungy. We disembarked. No one was around. It was quite, shady and cool. I loved the feeling. A mystic air sounded at the sight of animal bones and skulls interlaced in curious shapes and hung with soft vines outside the lean-tos and over stumps around* (Figure 2)*. They were fascinating and intriguing signs. Seven years later, I learned that they honored the jkyo aemo, hypostases of the hunted individuals. This is part of a much more complex hunting ritual and praxis. Respect and zeal to certain parts of the eaten bodies guarantee the return of the spiritual-selves of the animals back to the material world and thereby the constant replenishent of the prey populations. Returning to the canoe, we continued our journey with growing appeals and questions.*

**Figure 3:**
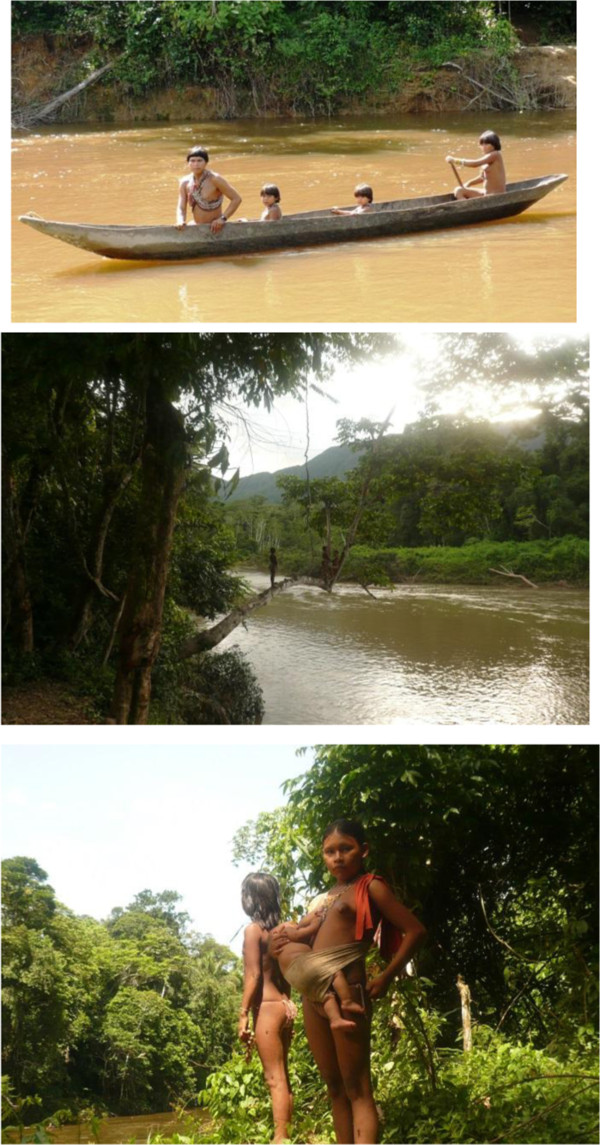
Stanford and I going up river with our dear Piaroa friend Basilio, Animal skulls interlaced, August 1995.

*An unpassable rapid flanked by huge trees impeded our transit and forced us to end totally the fluvial trip* (Figure 3). *It was good to walk. Entering the forest was like an open fresh Cathedral protecting our skin from the strong sun and embracing our steps. But we were totally lost. It was then, soon after we realized we had been walking around in circles when Jtobáappeared alone. He was tall and except for a loincloth and a few beaded necklaces, he was naked. His body’s profile, salient over the many tones of greens, daunted us. However, his big smile announced his friendly disposition. His feet were engraved in our memory: they were prominent, strong and curved inward, just as if they had been designed as perfect climbing tools. We tried our best to ask Jtobáto take us to his house. Months later we understood that he explained the best he could that we were taking the wrong trail. Probably he saw us since the day before and realized our ineptness as forest trekkers. He arrived at his community at nighttime after walking in circles for at least 4 hours.*

The first impression I had of the Jotï has persisted until today: gentle, smiling, resourceful, sensitive and determined people. The strong impact that their forest made on me has also remained. Both trigger my years of fieldwork, furthermore with increased knowledge of the forest and people of Maigualida augmented my admiration and respect for them; also my awareness of the huge range of differences among us humans as well as our likenesses.

This first trip lasted five weeks. Although we were lost much of the time, and disoriented among tiny rivers and huge trees, we managed to visit around 10 Jotï communities in different types of floral communities. We decided to write a project that allowed us to go back to the Maigualida: *Quantitative Ethnobotany and Behavioral Ecology of the Jotï Indians of the Circum-Maigualida, Amazonas and Bolívar States, Venezuela*. After getting the permits and some research grants, we started our ethnoecological fieldwork in 4 communities located in different ecological and social settings which were separated by 3-8 days of foot travel from each other. Further explorations, data collection and learning of the language was initiated in May of 1996. The range of similarities and differences were fascinating but also challenging. We were confronted with the task of making “scientific” order out of the apparent chaotic vastness of both ecological and socio-cultural settings.

### May 1996

*It happened during a season of heavy rains. We had been trekking in an exceptionally closed canopy and hilly rain forest in Sierra Maigualida with two Jotï brothers, Jani Jlado andAbado. We had met them a few days before and they agreed to take us to meet some of their relatives. Many shades of green covered our days and nights like an unbroken mat. We had not had a clear view of the sun or the sky for four days. Denseness weaved in an endless intensity of greens dwelling in such a huge diversity of plant life-forms stroked my eyes with a wonderful appeal. Tiny open spots filtered here and there little patches of light glowing into the tangled understory. Tall, straight, leaning, wide, twisted, curled, soft or hard stems and branches of trees, vines, shrubs, treelets and herbs, puffed-up to grasp the meager amount of sun that entered the forest. Greens were halted irregularly by colorful caterpillars, mushrooms and elaborate flowers, notoriously the brightly bracteolate bromeliads and orchids. Sneakily, heavy drops ofwater fell incessantly on each form, including us. Sun and water calibrate prominent dynamics in the Amazon letting unique lifestyles to be set into motion in the forest. Life takes on so many colors and profiles in the forest that biodiversity seems endless. Such a setting makes us, humans, just one of millions of material structures of existence. A very humble feeling is a constant mood in this setting. Humility reminds us constantly about our fragility and interdependence, in essence about our role in a chain of direct interrelationships with different species of which we are just consumers, unable to survive without recourse to many producers. Human interactions in the forest can be creative or deleterious, diverse and consistent.*

**Figure 4:**
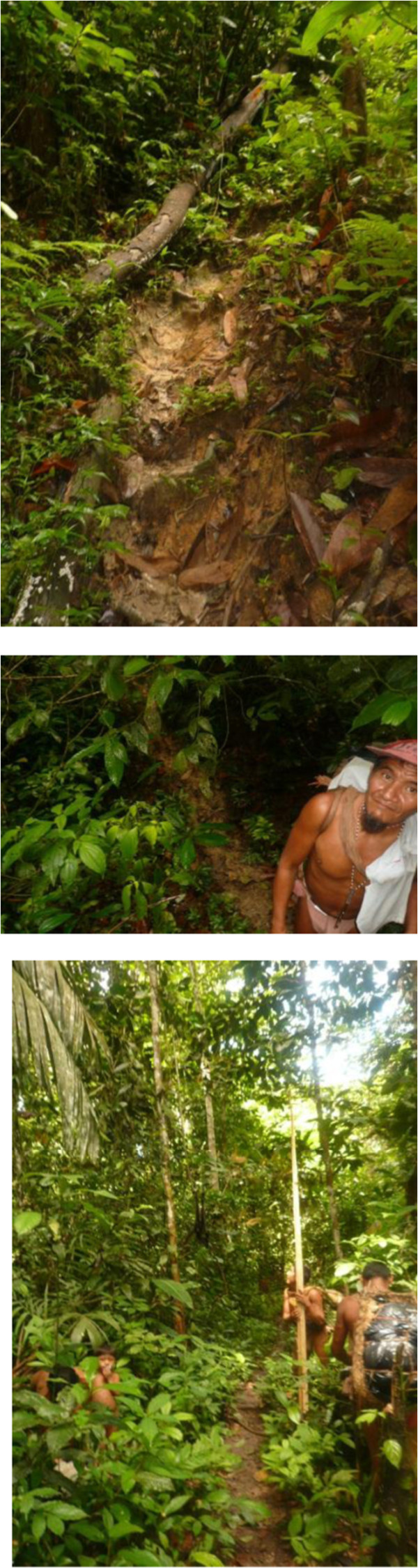
Typical Jotï trail.

*Climbing was a slow endeavor since the Jotï, with the aid of sharp machetes, had to open the trail for us to be able to go through* (Figure 4)*. The Amazon is life. Nothing remains still in the rain forest. All processes are dynamic. Life expresses itself in eternal movement. A freshly cut trail will stay open for only a few days. The extremely low densities of human population make it impossible to spare the labor needed to maintain the footpaths, especially when they are little used. Trails are prominent human disturbances. They activate dynamic ecological processes allowing light to enter in bigger patches and halting the continuity of habitats and niches. Sun-loving creatures fight to bloom in those open spaces. Creeping life-forms absorb the light in no time and turn it into new shades of life appropriating the shape of plants or arthropods. Pleased and gentle butterflies, bees and hummingbirds fill the clearings in the forest increasing pollinization activity by the hundreds which in turn set off the production of more and more life. Forest-life processes were here instigated by people. Our steps were silent and slow consenting a rather unique opportunity to sense the forest? We could hear the manifold sounds that speed hides and shut from our perception? For at least one hour we could make out the faint rumble of rushing water which grew louder with each passing step. Thus, we expected to run into a river, which in turn will substitute our sheltered ceiling of trees for a solitary sky tinted by hues of tropical blues. It was unexpectedly bright however, to reach a scene that had followed me as a pennant of unusual exquisiteness.*

The sudden appearance of massive light hurt my eyes. The sun’s rays entered the water reflecting vigorous life. A gorge appeared in plenitude. Misty low clouds levitated over the water in a continuum of states of liquid-vapor in a single essence. We could see the sun and the sky for the first time after four days. The tree’s stockades came to an end abruptly on both sides of the river’s bank. The bluest sky was competing in intensity with the reverberation of the water’s rapids. A band of yellow, blue and red macaws described a twisted arc on their sonorous playful fly contrasting with so much blue now. Big grey rocks covered with moss, lichens and fungi, stood out solid rather timid?, against the strength of the water, which permeated the atmosphere. A strong scent of humidity fluttered around. Away from some minutes?, I could feel how my body was penetrated by scents, sounds and lights seemly forever.

The beauty of the place minimized the real death threatening moment that we were about to experience. The trail was not halted by the river. Rather, in order to keep going ahead on our hike, we had to cross a log that had been cut down and stretched from one ledge of the gorge to the next, acting as a bridge. Although the log was huge, it seemed tiny in perspective against the rapids intoning its strength some 165 feet below. The portion of log overhanging the white water was about 50 feet long. It was wet and covered by moss and therefore probably slippery and dangerous to cross. But turning back was not an option; the evident consensus set silently our motion to cross the river? The Jotï helped us with our heavy backpacks. Dexterously and effortlessly, they crossed the log in no time and then they stopped and stared at us from the other side, waiting for us to come. Fearful that we couldn’t keep our balance upright, Stan and I had to straddle the log bridge, with our legs and arms wrapped around the trunk, and drag ourselves forward little by little. Stan went first. He was advancing firm and steady. I was very apprehensive while he was crossing the log. He was good and agile; his thin but sturdy body fit the embrace of the trunk. A new serenity invaded me when I saw him safe on the other side waiting for me. I followed him. My obvious clumsiness made the journey longer. I sat on the log. I felt my own fluids joining the wet spongy but pliable texture of the moss. For a few minutes my body grew used to the new texture. I glided and embraced the wide trunk. My heart bumped faster. A few more feet and I was in the middle of the log. I could not resist thinking about dying there. I closed my eyes and felt a profuse love, simultaneous with a deep gratitude. Thoughts of beauty and fear ran through me. I kept going forward. I slid twice more and my little hat almost felt once. I got scared but moved on more carefully. It has been the one time in my life when beauty seemed bigger than death in all dimensions. Mixing such different realms, aesthetics and transcendence, provided me with a new enchanted sense of life. Finally I joined Stan at the opposite end. We smiled and breathed a silent sigh of relief and accomplishment. All stared for a brief moment and aborbed the mixing of attractive danger and beauty concentrated in that spot. No word was said. I knew thereafter that I always belonged there.

*Ahead of us was an extremely steep trail. As if entering a tunnel, we crossed the threshold towards the green again. A shallow, shapeless forest made out of mostly monocots embraced us. High Strelitziaceae, gracious Cyperaceae attractive Marantaceae, colorful Zingiberaceae, attractive Acantheacea, scented Araceae and broad-leaved Musaceae, dotted here and there by Cecropiaceae and Piperaceae. The softer and shallower woods gave way transitionally to more sturdy forest. We were back under the shelter of greens. Tall Lecythidaceae, Burseraceae, Flacourticacea, Rubiaceae and Meliaceae flanked a more beaten-down trail, indicating regular human traffic. The almost vertical path took us to the first house we had seen in several days. It was sitting on a small open plateau enfolded by distant hills. It appeared bright. It was made from the palm leaves of ulu (Attalea maripa Mart.) from bottom to apex. A single opening acted as door and window. No one was around. Going up gradually, we had arrived at over 900 m asl. Our Jotï friends announced that we had arrived at Jkujkyo luwei (‘the house of a man named Jkujkyo’), and the surrounding area was known as Jkujkyo majaijka (‘Jkujkyo’s place’).*

**Figure 5:**
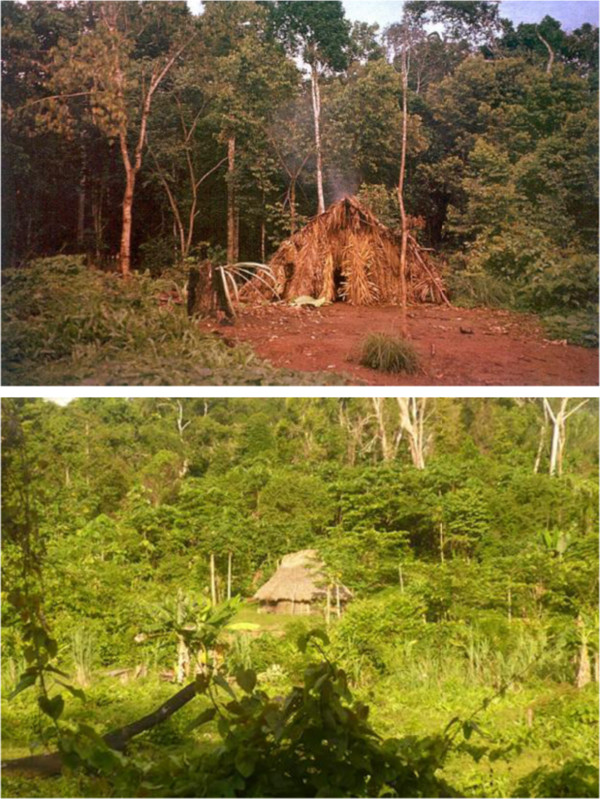
***Jkujkyo bajaila*&*luwei, 1996, 2012.***

*Jkujkyo bajailacame after strenuous walks* (Figure 5)*. Water was present all the time. Not only was it raining day and night, but we were constantly crossing many streams or the same spirally stream into which we submerged our permanently wet bodies. Our human fluids were confused and analogous to the forest substances. Jkujkyo’s house was an extension of the forest, damp, dark and occupied by indefinite creatures that often produced scurried sounds at night. Water dripped and poured through the flimsy and loosely thatched roof just like it did when we were out under the canopy. A non- conscious identity assured us security for finding shelter in a human-made house? We stayed there alone for several days, searching for Jkujkyo’s whereabouts and exploring the forest, old gardens, sunsets and sounds. We learned from our companions the process of making strings out of wild and cultivated fibers; we rested and gained some confidence in our acquaintance with the hilly rain-forest.*

Sometimes, the focus of memoirs clings attached to our mind, unconsciously, to beauty, happiness, desires, knowledge, transcendence or mysticism among many other abstract or concrete concepts and sensations. To me, the memory of *Jkujkyo bajail*a*’s* is associated with intensity to a day when beauty overcame death; within a punctual moment in my life where aesthetic and transcendence somehow were tied in an illogical but continuous ethos that undisclosed a little about the complexities of human nature.

## Alterity

### June 1996

**Figure 6:**
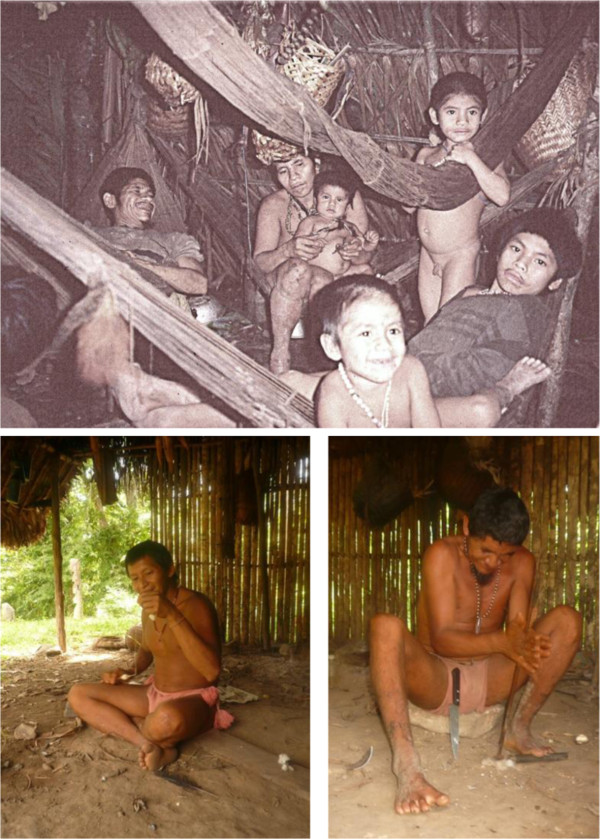
***Jkujkyo *and his family 1996*, Jlade *spinning cotton*, *2012*, and Bijkio starting a fire *2013b.**

*The day I met Jkujkyo a confusing blend of sensations of proximity and distance were pulling me in different and rushed directions through logical and sensual alleys. On the eighth morning of trekking, we woke up determined to keep on hiking deeper into the Sierra Maigualida, hoping to make contact with isolated Jotï bands. Although we had not come across any people yet, we had seen human tracks, houses, tools, gardens and even believed to have heard some notes of flute music. We were tired and hungry, since our food supply was close to nothing and thus we were anxious to find people. A little past noontime, a progressively more pungent odor bathed the air. I never had smelled a scent that strong before. As we walked through a forest with a rather sparse understory, the odor became stronger and closer. Swiftly we ran into the source of the odor: a small fireplace where ulu dale palm fruits (Attalea maripa* Mart.*) were being cooked. A Jotï woman accompanied by her four children, all boys, was attending the fire. Despite being so isolated and alone they did not manifest any surprise or fear upon our encounter. They were just curious like us. Maybe they were a little eager to explore who we could be. We approached the shelter slowly and without concealing some astonishment we drew near to the lean-to behind Jani Jlado andAbado. Few words were interchanged. Our guides were originally from an area close to there. They were familiar with the people and environment that we were crossing through. Without mediating a word, it was decided that we would camp there as well. Jani Jlado andAbado went to look for some leaves to build our own shelter for the night. We waited, smiled and looked around. We humans are diverse and pliable. I was captivated by the encounter and wanted to learn some more about the people we ran into, but we did not have yet have the skills to communicate effectively in their language. All of the Jotï present, including our guides, were monolingual. Gestures, then, worked as a good tool to convey meanings* (Figure 6)*.*

The woman seemed to be in her late 30′s, she was of medium contexture, about 5′ and had a friendly smile. As most Jotï woman I had met, her only attire was a small cotton loincloth. She, as well as her children, was profusely adorned with long necklaces made out of wild and cultivated beads, but also mixed with pieces of bird’s bones and feathers, mammals’ dentures, pieces of holed canes and tins, old hollowed coins and used thread carrels, and a few glass beads. Two necklaces covered all her breast, used laterally and crossed in a croix that met just under her breast. She had also thread carrels inserted into her earlobes. Disordered black dots of a thick paint covered part of her arms and legs. Her kids, ranging between about 2 and 9 years old, smiled permanently and were using similar black dots on their bodies but fewer beads.

A peaceful air ran without hurry. Silently we just observed them and they observed us.

Ulu fruits are single seed ovals of about 2-3 cm in diameter surrounded by edible meat or mesocarp. Heavy racemes of the fruits are collected and cooked directly without processing in boiled water. It is highly appreciated throughout the Amazon region as a good source of protein and lipids. Huge piles of eaten seeds rested high to the sides of the lean-to made from the same palm tree’s leaves as a shelter under the canopy. It was clear that they had been feeding generously from ulu fruits. Judging from the amount heaped in a pile, they had probably been there for about a month having this fruit as a staple food. It emits a particularly strong, unmistakable scent that forever was sealed in my memory as the hungriest days of my life, but also filled with strong emotions and happiness. It prompted also the possibility of considering the Amazonian Indians as builders of the forest through the creation of seed banks and dispersal of seeds. We have been endorsing this idea also over the years.

*Jani Jlado andAbado came back with long leaves (Heliconia spp., Attalea spp., Phenakospermun guyannense* (L.C. Rich.) Endl. ex Miq.). *Simple knots were rapidly and laxly weaved from their midribs. A large load of thin poles of different species (Cecropia *spp., *Apeiba* spp., *Ouratea* spp.) *were also brought to frame the structure of our make-shift shelter. Soon we had a lean-to with enough poles to hang our hammocks. We were busy trying to make a home for ourselves when we heard a soft whistle produced by a flute made out of a hollowed thin cane. We looked up and there was Jkujkyo, the husband and father of the people we had interacted with for the last couple of hours.*

Jkujkyo arrived with his son of about 13 years old. Jkujkyo had a broad smile and was muscularly built. He talked and walked very fast. His human fluids emitted a scent strongly mixed with the forest odors.

We spent a couple of days and nights among Jkujkyo and his people. It was pleasant. We walked and explored the area, but also rested. Sadly we were not able to collect any samples since we were already carrying heavy loads and had very little or no food at all. Jkujkyo’s people shared with us their food, mostly ulu fruits, raw and processed as soups and juices. Morning, noon and evening ulu was the staple food. I found that ulu fruit tastes like soap and its scent is associated in my sensual memory with being amazed and alien as ever as well as being observed and studied closely by very different people.

The third morning we were ready to continue on with our journey and set out to walk after dawn. It was a calm sunny morning. Parts of the trail were well kept indicating more frequent use. After just about three hours walk through a relatively easy but steady climb, we arrived at a quiet handsome settlement: Abiema’s community.

Abiema was a natural leader among the Jotï. The settlement was located at the top of a hill (~1000 m asl) flanked by bigger mountains. His house looked strange because it was unlike any other Jotï house we had seen up to that point. It was rather well made, sturdy, larger, round and tall, in fact its height appeared to be greater than its diameter.

Upon looking at him, I felt an immediate attraction to the image that he inspired in me: that of unspoiled primitiveness. Unavoidably, at first impression I objectified him. In the second that I discovered my own archetypal projection I realized more similarities than differences between him and us. Abiema means the bearded one. He, like Jkujkyo, had a short black beard. Not he nor any of his group were using at that time any necklaces, which I found curious. Years later we learned that donning no paraphernalia or adornments such as beads is part of the mortuary ritual. Probably this group was honoring the death of a former member of the community and that could explain why their bodies were just covered by the traditional annatto-dyed loin-cloth.

**Figure 7:**
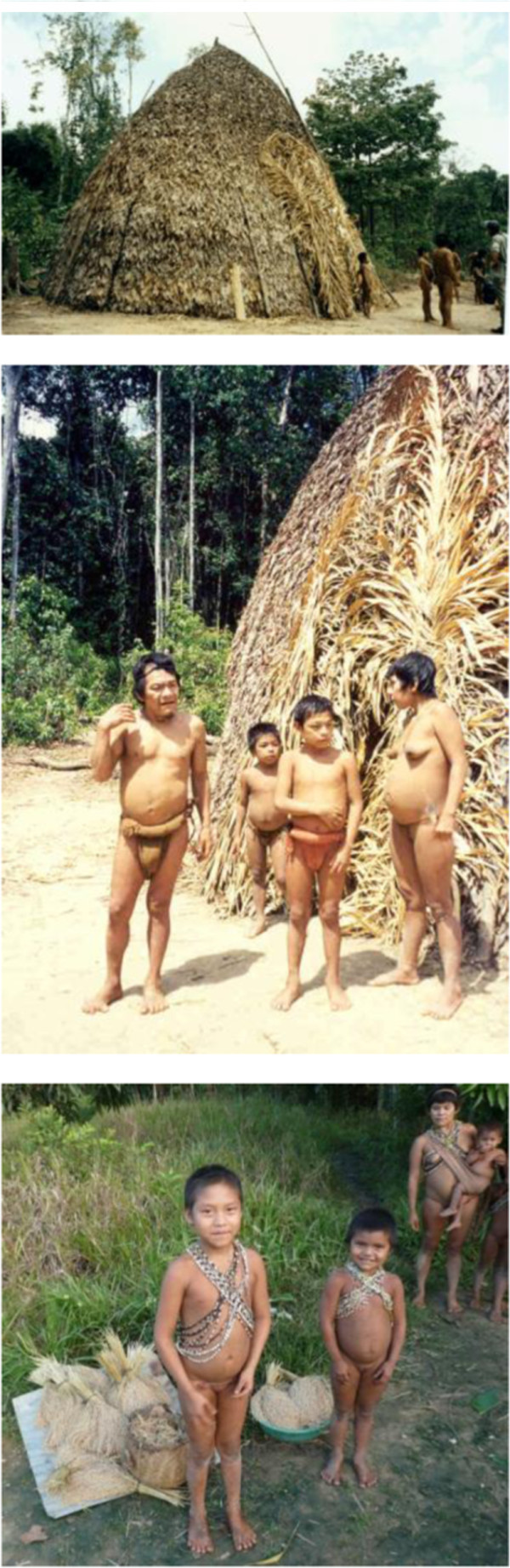
***Abiema jluwe *and his family 1996, some of his descendents 2011.**

*There were about 20 people in Abiema’s group. He had two wives. Instead of the cotton loincloth, the younger one was wearing a monocot leaf fastened onto a strip of jono jyeï’s bark (Lecythis* sp.*) circling her waist, thus indicating that she was having her menstrual period, therefore was potentially dangerous and not allowed to cook. The elder one was carrying a baby. They did not show any fear upon seeing us* (Figure 7)*. I had a twinkling sensation that they had seen us during the last few days approaching their lands. They were friendly.*

Abiema exuded a sort of dignity that I have sensed just a few times in my life. It was his certitude of being an honest, kind and comprehensive man. He seemed to be proud of being right where he was and who he was. He was a sturdily built man with broad shoulders. He smiled a few but meaningful times. His feet, but specially his big toes were particularly pointed inward, which I took to be a clear sign of climbing trees since his early years.

**Figure 8:**
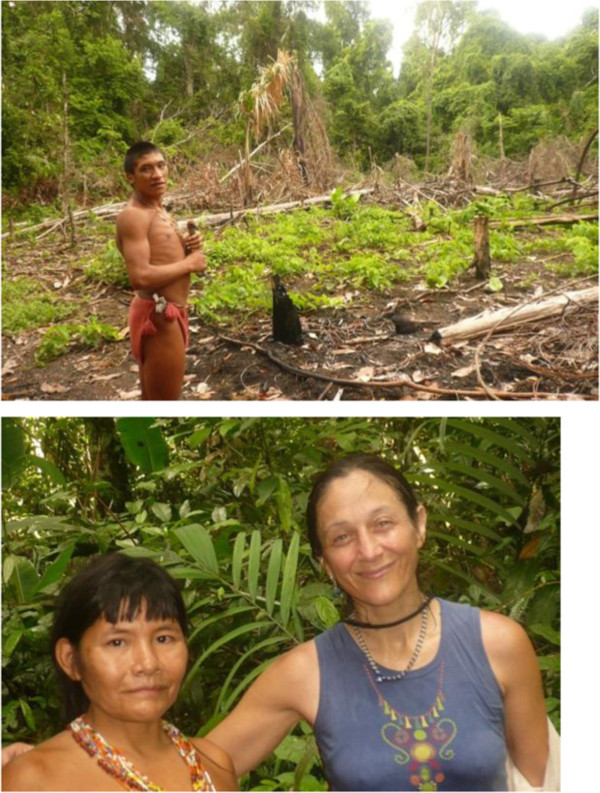
Hunting for Jotï gardens 1996, 2012.

Abiema and Jkujkyo were unique persons within mine as well as their own cultural landscapes. In more than one sense they were marginal to their culture. Their distinctiveness stemmed from several sources. They were settled in an isolated area on the hearth of Jotï cultural meanings and dynamics.? However, gradual waves of migrations turned this area, known as *Jwanëbo jedä* into a demographic sink area. Only a handful of family groups remained fulltime in this area. Jkujkyo’s and Abiema’s homes were located in *jwana jkuwë bajaila*, below the habitat of *jwana* (*Arthrostylidium* spp.). Jwana is a polysemic term that refers to a bamboo-like plant, to the hollow cane obtained from this plant and which is the primary raw material used to fabricate blowguns, and to the blowgun itself. The Jotï are blowgun hunters, thus periodic expeditions in search of the cane are carried out on a frequent basis by Jotï from all communities. *Arthrostylidium* is a grass with a very restrictive geographical distribution usually at the highest extremes of the tepuy province vegetation and on particular headwaters of a few rivers. Jkujkyo’s and Abiema’s groups acted as symbolic custodians of the jwana. Their communities moved constantly in a ratio of about 20 km. Their highly mobile houses are located at the base of the *jwana* region (900-1100 m asl). Since Jkujkyo and Abiema were guardians of the route towards the *jwana*, they had access to the flows of people from all Jotï communities. Therefore, despite being “geographically” isolated, they were probably among the liveliest social communities since they were in the capacity of passing information and services through many within their ethnic group. This ethnic bridge transcends the material sphere. Jkujkyo’s and Abiema’s groups were also main holders of the *baede baJotï bajadï bajawa*. That is they still carried on the traditional Jotï lifestyle, the one rooted in the ancestral ways of being and acting in the world. Not surprisingly therefore, Jkujkyo and Abiema were actually feared by many of their fellow Jotï of different communities. They were ascribed magical and shamanistic powers that we were able to feel but not see in action. Later, I heard that Jkujkyo’s house had been burned down on one occasion in a bigger community by accusations tinted with witchcraft and malevolent spiritual powers. Meeting Jkujkyo’s and Abiema’s groups provided me with a broader notion of human nature or with the feeling that they had a us (Figure 8).

## Relativity

Despite the small total population size of the Jotï (< 1000 persons), we were amazed at the huge diversity within the ethnic group from the first contacts?. That fact prompted us to select four communities for our study in the attempt to capture some of this variation in social, cultural and ecological spheres. These three spheres turned to be intertwined and relative to each other in many contexts such as the following reminiscence portrays.

### July 1996

We had spent the last month at Majagua balo, a relatively recently founded Jotï community of about 25 people. At about 250 m asl, both the local biotic and sociocultural characteristics are different from higher basi-montane and sub-montane areas (600-1000 m asl) more common to the Jotï biocultural landscapes. The community was settled on the left bank of a narrow and winding creek that flows into the Parucito River. During the rainy season it takes a whole day to make the trip upriver in a dugout canoe powered by an outboard motor from the mouth of the river to the Jotï community, whereas this same trip takes about 3 to 4 days in the dry season when the water level is much lower. According to their oral traditions, the Majagua people had migrated to this zone from the North about ten years before. While building strategies to understand and deal with new configurations imposed by contact with other indigenous groups (mainly Eñepa, Piaroa and Yabarana)as well as Westerners, the Jotï had been colonizing new environmental (lower gallery and flooded forests) and social settings.

Although the Jotï at Majagua were quite friendly and hospitable, we were ready to explore some other Jotï settlements in this area. Uli au balo [big water land], or the land of Abie jolowajka Jotï [the coarse bearded people], seemed like an appealing place to explore as it was the home of a very intriguing subgroup. Although they were said to be located just a day’s walk upriver from where we were, all Jotï at Majagua were reluctant to take us there. They were very apprehensive of the Abie jolowajka Jotï, who were described as being powerful witches, trekkers without gardens, hostile and strong-willed people. Such remarks only served to arouse even more our eagerness to visit that community and meet the people.

Marco, an Eñepa man from Culebra, a community located about six-hour walk awayfrom Majagua, was the only one inclined to take us to Uli au balo. He was married with a Jotï woman, a close relative of the Majagua people. Socially and structurally, he was clearly marginal to his own Eñepa people. Marco’s manners and movements seemed a little awkward, but we had no other option.

At sunrise of a soft morning we started walking towards Uli au balo. Marco was leading our way. The forest was damp, bursting with day-loving creatures. The manifold sounds echoing through the understory called and observed us. Butterflies, macaws, caterpillars, quails, wasps, hummingbirds, termites, squirrels, toucans, frogs, parrots, spiders, oropendolas, mosquitoes, orioles … so many life forms produced a non-tuned concerto decidedly exquisite and mysterious. Around the vines, trees, shrubs, palms, the sounds were not shy; they were statements, announcing the presence of those that produced them.

The trail was wet under the canopy; the sun rays did not touch the soil. The aroma of dampness permeated everywhere. Mushrooms and fungi sprouted profusely, cutting the profile of green here and there. Many tracts of the trail were muddy; some were even underwater, forcing us to literally swim to keep going forward. I discovered then that my backpack floated. It had been raining for the last couple of weeks and water accumulated and moved freely all over the forest. The rivers were clogged with so much water and no place to run off. We zigzagged across the same waterway at least five times, an uneasy task, given the slippery nature of the logs that serve as bridges in Jotï land, although at times, the bridge-log was itself underwater. Patches of the trail were closed by a thick curtain of tangled vines, trees, shrubs, understory, forcing us to hack it open with a machete.

We walked steadily but quietly behind Marcos for about 9 hours before reaching the community. At least half of that time it was pouring down rain. Our own sweaty fluids mixed with the warm water filtered from the sky to the screen of trees to land in our bodies. Our packs were also soaked and the extra water made them feel heavier. Crossing water bodies, big and small, moving and stagnant, was the constant image of our walking day.

At one point during the walk, Marco suddenly stopped for what seemed like a long time while bending over and peering intently at the ground. He indicated that he had momentarily lost sight of the trail and was trying to pick it up again. We thought it was quite odd the way he tilted his head so close to the ground. Then it dawned on us: he was almost blind. But just when we started to openly lament our predicament (¨the blind leading the blind¨) he found the path and set off again, and we had no choice but to follow. Finally, we glimpsed two small houses almost hidden under the canopy, one besides the other sheltered by the trees without a clearing evincing their presence from the sky. Although the trail was not an easy one, we realized that the distance itself from Majagua to Uli au balo was not so great. The obstacles to get to the community, mostly water in its many dispositions and modes, made Uli balo seem far away. As soon as we got there, people at the community came up to look at us. Two adult men and three women, a teenage boy of about 12 years old, four children and a baby girl were staring at us, aliens coming from nowhere. We experienced one of those astonishing moments that sediment in your memory forever. In this case, it was the absolute awareness given in a second, of being very different in a continuum of resemblances.

Stan’s field notes capture the strong experience: “For the first 10 or 15 seconds I got the deep impression that this was a quite isolated group of people that do not have hardly any connection with the outside world that I am used to. This is the sort of primitive ‘other’ object and experience that I desired for, that drove me to go into anthropology in the first place. Here it is right in front of me, looking back at me. Then I got this intense feeling: so what? What is so great about this? You cannot become one of these people yourself, you may never see them again, and maybe I should not even be here”. I had a similar sense. All our muscles and bones were exhausted, which probably enhanced the encounter and made it stick with me as a special state of mind-body; embraced by a magical halo of orange-brownish light emanating from a mixture of sunlight, water and clouds in the late afternoon forest. The longing and feeling evaporated soon due the preeminence of material needs. We turned all our attention to practical matters. It was going to rain again at any moment and we needed to make a shelter for the night. No word was pronounced. The whole community was staring at us while we frantically and hastily erected the poles and then the roof. They did not move one finger to help. The older man, whose name we learned later was Aunë (meaning ‘caiman’), had a quizzical smile on his face. He seemed to be studying us. Meanwhile the younger man looked intently at our possessions.

This is perhaps the strongest interaction I experienced among unknown Jotï without involving any words at all. Still, so much was interchanged among us. In my hammock that evening while listening to the water dropping onto the Attalea leaves, my thoughts wondered about how much relativity of perceptions taint the categories and behavior among us humans: the people that seemed to be the most “primitive” to us were actually the “vanguard” among “other” Jotï groups, and “all” the Jotï groups did not consider themselves as “one ethnic pack”.

Words came the next morning. We were ready to explore around the very muddy, swampy, flooded and damp surroundings. Since a research goal during this phase was to collect data related to the size, composition and distance from the settlements to the gardens and hunting areas, we asked the people to take us to their gardens. All of them stated insistently that they did not have a garden. Where do the plantains they were eating come from then? “You must have a garden!” we thought. Resolute, Stanford pleaded with them to show us their gardens. Finally they answered, “Jotea ñaña, ñajti jtojto dekae bada” or “yes, we have one but is really far away from here”. Our previous experiences with the expression, ñajti jtojto, seemed to indicate it meant the opposite of jamena dekae bada ‘close, nearby’. Furthermore, it was obvious that they were benefitting, at least partially, from garden products. We assumed that it was not that far away after all. So despite their reluctance, we convinced them to take us to their garden. The minute we set out to walk it started raining. It was that style of rain that exists just in the rainforest, hitting hard any form that avoids its speedy march. Water was flowing through all interstices. Water was penetrating shapes and life forms. Water was chanting to plants. Water was feeding life. All seemed to explode with the rain in the forest, now chaotic then diverse.

The trail to the garden was non-existent. We strode through the forest literally “running” behind Marco, who was also straining to kee up with Aunë and his teenage son. The boy seemed to enjoy our clumsiness and our huge effort to not get lost. It was very difficult to keep up with their speed. Their agility contrasted sharply with our ineptness, their belonging to the forest stood out from our clear otherness. After less than an hour, we ran into a temporary lake within the forest created by the accumulation of water. The Jotï were already emerging onto the other side of it and couldn’t care less about our whereabouts. In a desperate attempt to not lose our Jotï travel companions, we anxiously dove in and swam in their direction as fast as we could. Marco refused to enter the water, despite the fact that we were already as wet as possibly could be. He declared that he had had enough and he left us right then and there! He turned his back and without saying any more words he took off!

There we were … alone. Water was everywhere, in us, pouring endlessly from above and accumulated everywhere down below us. We could not even hear any other sound but water. All smelled of water. No time to think or complain. Swimming the best we could, we reached the lake edge and ran as fast as possible to catch our fleet Jotï “guides”. Our hearts were bouncing hard. The rain receded a little bit. We were able to hear the Jotï and “happily” ran behind them for the next two hours before reaching the garden. It was certainly ñajti jtojto dekae bada! We had been running, not walking, for the last three hours!

As a double gift, the rain stopped just as we reached the garden. It appeared to be a “semi-abandoned” garden; opened may be 5-7 years before. We spotted old patches of cultivated yam and sweet potato. Diverse varieties of standing banana trees were still productive. Happy to find food, we ate lots of bananas with pleasure and enjoyed a few warm, smiling rays of sun. Life seemed quiet again. We quickly set up a couple of sample plots and censused the crops inside them. Then we measured the perimeter the best we could and tried to get a GPS location reading, a major piece of data to plot our findings. The GPS was so wet that it did not work! Even though it was advertised as being waterproof. But this was no place to make a complaint…perhaps the Magellan company made it with non-tropical waters in mind… all the trouble to collect data that could not be georeferenced…

We waited for at least an hour while the GPS was taking a sunbath. After a while we tried again. It did not even catch one satellite! Our Jotï friends were getting impatient and told us they were leaving. Without dragging our feet, but lacking any option whatsoever we moved at their momentum and wishes. Running behind them on the way back was even harder since our bodies were exhausted and we knew it was a hard trail. We arrived at a place where a huge log was completely blocking the “trail”. Not only was it almost as tall as I am but it was also very slippery. Dexterously the Jotï went up and down within a few seconds and keep striding on down the trail. I clambered ineptly and fell. It was unfortunate and inopportune, but foremost it was very painful. Stanford shouted for help. Much to our surprise, the Jotï stopped and looked back at me. They managed to try a smile but could not understand why we were calling them. Stanford showed them my awful state. They just stared at us with an empty but also unannoyed look on their faces. Beyond this visual contact, there was no communication at all. Two very different ethos and codes of human relations were side by side in this event: parallel but not inter-communicable lifestyles.

Struggling with hunger, pain and mental weariness, we realized suddenly about their absolute lack of empathy towards us. Our fear about getting lost, was not comprehensible to them. Adults our age in their culture have already mastered the basic skills and knowledge about the forest. Therefore they are completely apt to survive alone without requesting help. Our claim was totally new to them. They could not believe our incapacity to find our way without their assistance. The teenager’s playful way of disappearing and then reappearing gaudily from our sight while we were rushing to keep up with them, may have been just a gesture of friendship and a way to test our humanity, but I do not believe that he meant to ¨lose¨ us or harm us in any way. We learned that empathy does not match survival skills in the forest.

Water started to fall again. Further reflection was postponed while we had to keep on walking. I clambered over the log with Stanford’s help, took a stick to walk again and after a little while I was running for survival (again).

Water was still pouring from the sky when we arrived contentedly back at the community where the two houses were surrounded by flooded patches of forest. No word was said again. A mixture of feelings and ideas kept creeping to my mind all through the walk. Leading my thoughts was the certitude of the relativity of notions as time and space, of possibilities to communicate beyond language and to interact in deepness despite having just one event to recall. The complex of space-time is tightly tied for people like the Jotï. Forest space, its dynamics and functioning constitute major tools for this people. Time is a measure of dynamics in space. Human’s interactions are not independent of the forest. They learn to be with and in the forest. Our inability to see the forest life forms, its structure, its processes and eternal changing state is our handicap; however it comprises an important survival kit for them. Knowledge about the forest and themselves is a cumulative process that each Jotï acquires since they are born. In the end, knowledge is built more through interactions than by pure intellectual reception and processing. Life is knowledge.

Two years after this event, *Majlu* arrived at a Jotï community where Stanford and I had been staying for a while. His two and a half year-old daughter was very sick and needed emergency medical help. We decided to take them to the closest place where Western medical care was available. In the dugout canoe and walking, Majlu was carrying his girl in a most tender way, providing water and love every second of the way. This man, considered a strong and non-sensitive Jotï, has been the only Jotï that I have seen crying profusely, facing the impotence of alleviating the pain of his dying little girl. Her distended belly evidenced many parasites living in her bony body. But no food would stay in her system. She vomited permanently everything, and even the multivitamins that we gave her for a week were finally discarded. She was very ill, extremely thin, weak and almost unconscious. It was very sad to see Majlu caressing the girl and in so much despair silently. We arrived at the closest dispensary on the 3rd day at 5 p.m., located in an indigenous multi-ethnic community of around 100 people. It was a little old hut, with mud walls, dirt floor and broken ceiling. The only sign it was a health clinic was a new weighing-scale. The afternoon was giving way to the night in the forest and many insects were singing in early March 1998. The nurse, a Piaroa male, gave the girl a shot but in less than an hour she passed away. Several Yabarana women bawled loudly and steadily while Majlu changed his expression from sadness to alertness and immediately declared: “I am leaving now”. He ran fast and took off in the middle of the night, leaving us the corpse of his daughter. Surprised, local people and us carried out a minimal but respectful burial ceremony. The women wailed all night long taking turns, some children cried, and a Yekwana man made a wood coffin. Despite being very sad, we tried to figure out the different notions and behavior towards the dead girl. No one, except us, found it unusual that the only relative of the deceased departed the minute after she died. Everyone took on the mortuary ceremony as a community effort and duty despite that nobody there had even ever seen Majlu or his daughter before. No one questioned why she died for lack of medical help since the only real disease she had was too many parasites. The next afternoon after the morning burial, no one cried again and everything was back to normal. We also took off.

**Figure 9:**
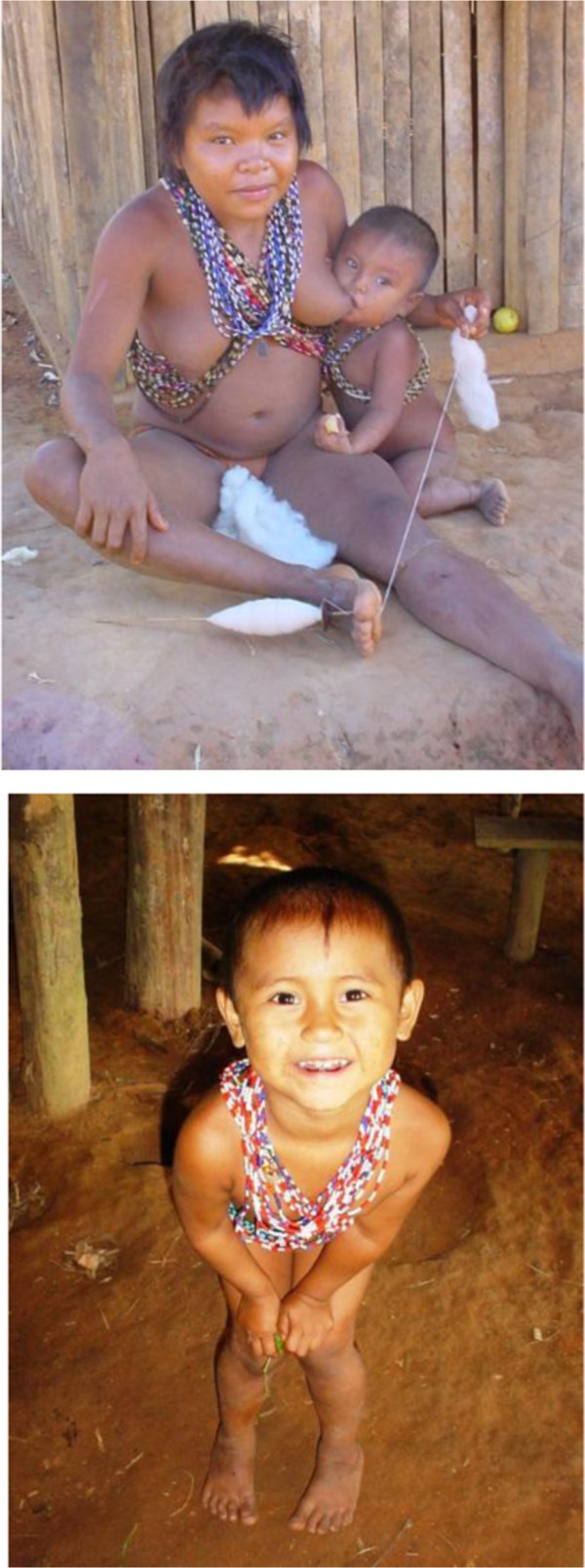
Jotï high morbility and mortality is confronted with high natality.

Sadly the Jotï have high morbidity and mortality rates, most of them associated to infectious diseases easily avoidable with proper care (Figure 9). Over the years we have learned that they have three etiological explanations of illnesses: autochthonous, introduced, and the synergy of both. They have good ways to cure their own “traditional” illnesses but not the foreign ones, those usually fatal for them.

## Final words

I assume the risk of being pigeonholed as dialectical, symmetrical or a-theoretical. However, my own definition of Ethno-ecology settles me at home in a no-space or nobody quarter, while simultaneously it touches virtually all human endeavor nowadays: global warming and delicate cognitive process. Amid anthropology and ecology, sociology and botany, philosophy and zoology, ethnoecology is difficult to grasp as a single discipline. Defining ethno-ecology as inter- and trans-disciplinary is just a piece of the multiple facets of this realm. Ethno-ecological researchers find themselves often dwelling in dilettante stances of immanent liminal states. Liminal spaces present an endless supply of surprises, explorations and enthrallments to me. I identify myself with interstitial areas, where life can be stagnant sometimes, but can exploit more often?. In-between disciplines and cultures, in the middle of diverse theories and anomalous methods, inhabiting alien spaces while discovering many similarities to their hometowns in there. Usually it is more difficult to find the right context to explain how to enter the quotidian lives of communities different than ours while simultaneously we try to know their local biological diversities, understanding their interactions and dynamics.

I speculate that as many anthropologists [20], also some ethno-ecologists openly or covertly, indulge themselves in a self-reflective chase for answering ontological questions while conducting their research [21]. In fact, the geographical setting, the ethnic group, the theoretical and methodological backbones of a project depict an intimate self beyond the investigator in charge of a project. The first time is probably a non-conscious engagement of the ethno-ecologist with him or herself to a socio-ecological setting often times alien to her/him.

## Competing interests

The author declares that she has no competing interests.

## References

[B1] Tyler S James C, Marcus GE Post-Modern Ethnography: from document of the occult to occult document Writing culture: the poetics and politics of Ethnography 1986 Berkeley: University of California Press

[B2] Zent E Etnobotánica Hotï: Explorando las interacciones entre la flora y el ser humano del Amazonas Venezolano 1999 Ahens: Tesis de PhD University of Gergia xxiv, 519 pp

[B3] Zent E Ecogony J Venezuelan Amazon Environmental Research Letters 8 2013 015008. http://stacks.iop.org/1748-9326/8/015008

[B4] Bodin R Finding Brown Gold 1969 27 12 7 9

[B5] Bou J Found … But Lost Brown Gold 1970 28 3 6 7

[B6] Dye P Yowana contact Brown Gold 1970 28 8 3 5 15

[B7] Jangoux J Observations on the Hoti Indians of Venezuela 1971 Manuscript

[B8] Eibl-Eibesfeldt I Die Waruwádu (Yuwana), ein kurzlich entdeckter, noch unerforschter Indianerstamm Venezuelas Anthropos 1973 68 1–2 137 144

[B9] Corradini H Los Indios Chicanos Venezuela Misionera 1973 XXXV 405 6 9 (406):42-44; (407):88-91. http://www.academia.edu/501293/Etnobotanica_Cuantitativa_entre_los_Hoti_Selva_Tropical_Guayana_Venezolana

[B10] Guarisma V Los Hoti: introducción etno-lingüística Tesis de Licenciatura 1974 Caracas: Universidad Central de Venezuela

[B11] Coppens W Philippe M Les Indiens Hoti: compte rendu de missions L'Homme 1974 XIV 3-4 131 14223930277

[B12] Coppens W Contribución al estudio de las actividades de subsistencia de los Hotis del río Kaima Boletín Indigenista Venezolano 1975 XVI 12 65 78 nueva época21216839

[B13] Coppens W Coppens W Los Hoti Los Aborígenes de Venezuela 1983 II Caracas: Fundación La Salle/Monte Avila Editores

[B14] Guarisma V Coppens W Vocabulario Hoti Antropológica 1978 49 3 2723914511

[B15] Vilera D Introducción morfológica de la lengua Hoti 1985 Caracas: Tesis de Licenciatura, Escuela de Antropología, Universidad Central de Venezuela

[B16] Huber O Alarcón C Mapa de vegetación de Venezuela 1988 Caracas, Venezuela: 1:2.000.000. Ministerio del Ambiente y de los Recursos Naturales Renovables and The Nature Conservancy

[B17] Huber O Rosales J Berry P Huber O, Rosales J Estudios Botánicos en las Montañas Altas de la Cuenca del Río Caura (Estado Bolívar, Venezuela) 1997 Ecología de la cuenca del Río Caura, Venezuela: II. Estudios Especiales. Scientia Guaianae 441 468

[B18] Yerena 1982 voucher collectionsy Herbario nacional de Venezuela (VEN): Herbario Víctor Manuel Ovalles (MYF)

[B19] Fernández 1980-1985 voucher collectionsy Herbario nacional de Venezuela (VEN): Herbario Víctor Manuel Ovalles (MYF)

[B20] Rosaldo R Ideology, place, and people without culture. Place and voice in anthropological theory Cult Anthropol 1988 3 1 77 87 10.1525/can.1988.3.1.02a00070

[B21] Nolan J Pieroni A Recollections, reflections, and revelations: ethnobiologists and their “First Time” in the field J Ethnobiol Ethnomed 2013 9 12 10.1186/1746-4269-9-12 23425433 PMC3599945

